# Supplementary material for: Role of the Redox State of Human Peroxiredoxin-5 on Its TLR4-Activating DAMP Function
Source: Antioxidants (Basel). 2021 Nov 27;10(12):1902. doi: 10.3390/antiox10121902 (PMC8750366; doi:10.3390/antiox10121902)
Supplement: Supplementary file 1 [file antioxidants-10-01902-s001.zip › antioxidants-1484517-supplementary.pdf]

Supplementary Information for

Role of the redox state of human peroxiredoxin-5 on its TLR4-activating DAMP function

Mégane A. Poncin, Pierre Van Meerbeck, Joshua D. Simpson, André Clippe, François Tyckaert, Fabrice Bouillenne, Hervé Degand, André Matagne, Pierre Morsomme, Bernard Knoops, and David Alsteens

This PDF file includes:

Supplementary Figures 1 to 8

Supplementary Tables 1 and 2

Supplementary Materials and Methods

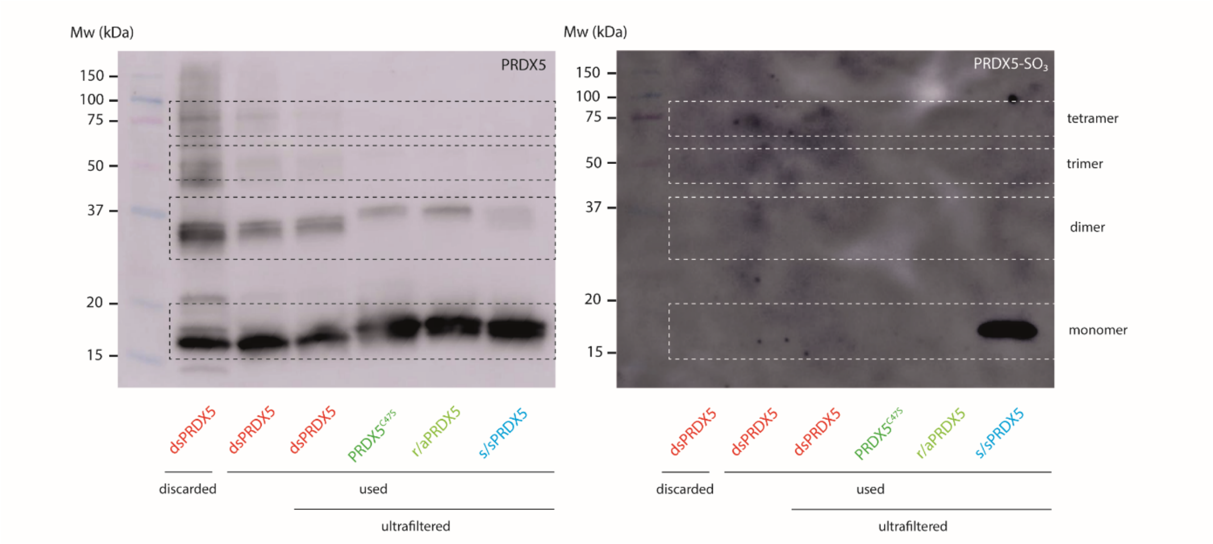

Figure S1. Western blot analysis of the PRDX5 redox forms performed under non-reducing conditions. Western blotting against PRDX5 (left) or against PRDX5-SO_3 (right). dsPRDX5 was probed before and after ultrafiltration. Representative of three independent experiments.

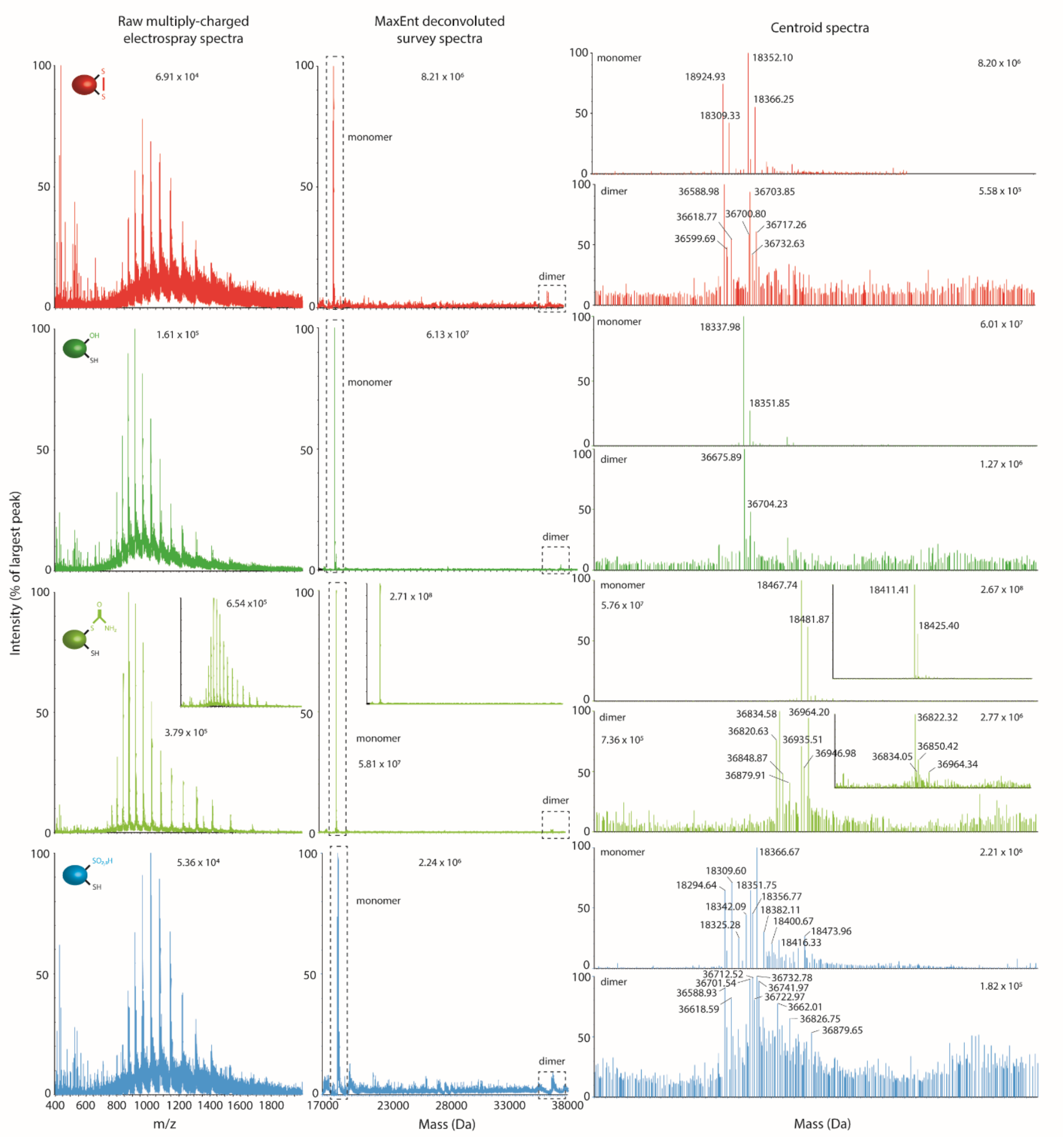

Figure S2. Direct-infusion ESI-Q-TOF analysis of the PRDX5 redox forms. Raw multiply-charged electrospray spectra (m/z 400-2000, left) were processed with MaxEnt1 to produce true molecular mass spectra (middle), from which were generated centroid spectra (right). Ranges of centroid spectra are 18,000-18,600 Da for peaks related to monomeric species (right, upper), and 36,200-37,200 Da for peaks related to dimeric species (right, lower). Potent Cys sulfenic acids were trapped with dimedone and remaining thiols were blocked with 2-chloroacetamide (CAM). Identification of Cys alkylated with iodoacetamide (IAM) in r/aPRDX5 was performed without using CAM prior to MS analysis (insets). Intensities and masses of the major peaks (≥20% and ≥40% in intensity relative to the largest peak of monomeric or dimeric species, respectively) are detailed in **Suppl. Table S1** (monomeric species) and **Suppl. Table S2** (dimeric species).

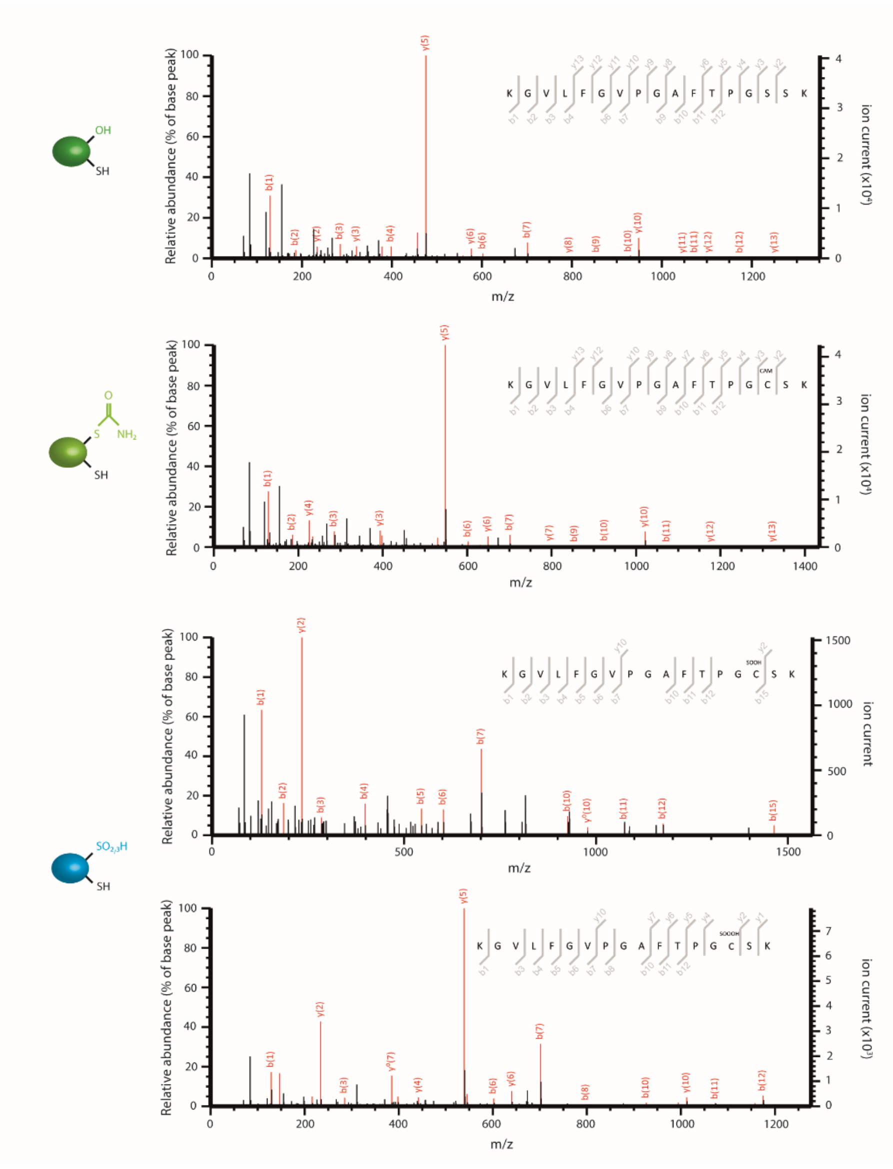

Figure S3. Representative spectra of fragments corresponding to peptides containing the 47th residue. Spectra showing the point mutation (C47S) in PRDX5^{C47S}, or the Cys redox modifications (*i.e.*, carbamidomethyl group in r/aPRDX5, or sulfinic acid and sulfonic acid in s/sPRDX5). Representative of three independent protein treatments. See **Supplementary Materials and Methods**.

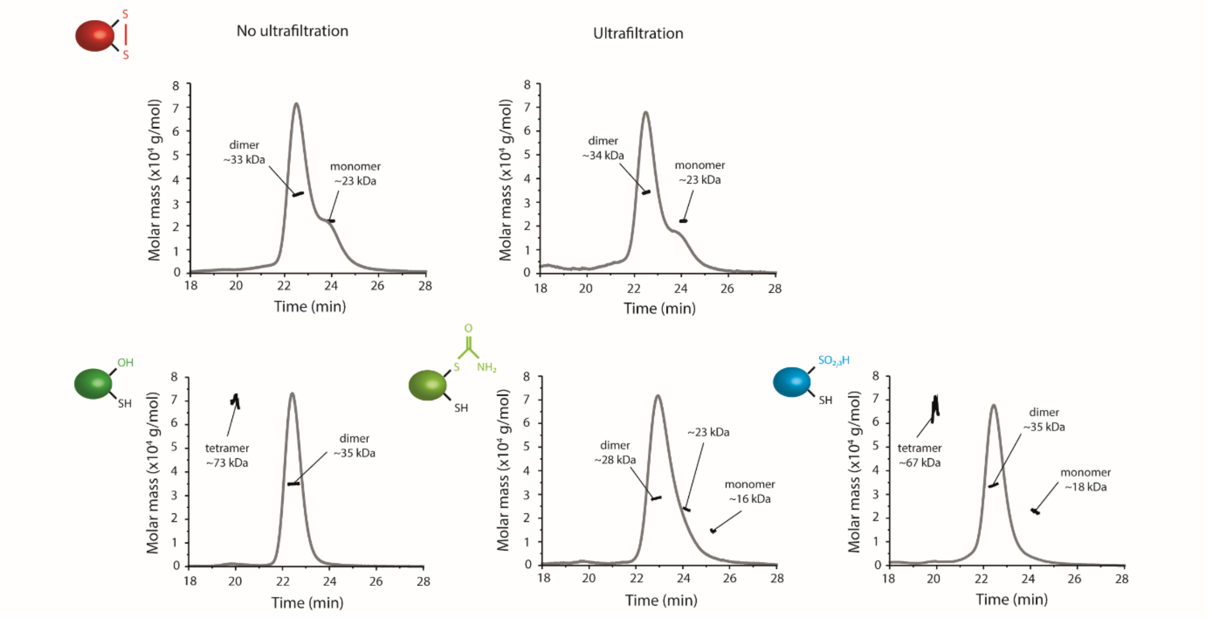

Figure S4. SEC-MALS analysis of the PRDX5 redox forms. Light scattering spectrum (grey line) of dsPRDX5 before (upper left) and after (upper right) ultrafiltration, and PRDX5$_{C47S}$ lower (left), r/aPRDX5 (lower middle), and s/sPRDX5 (lower right) allowing molar mass determination (black line) of the monomers and oligomers.

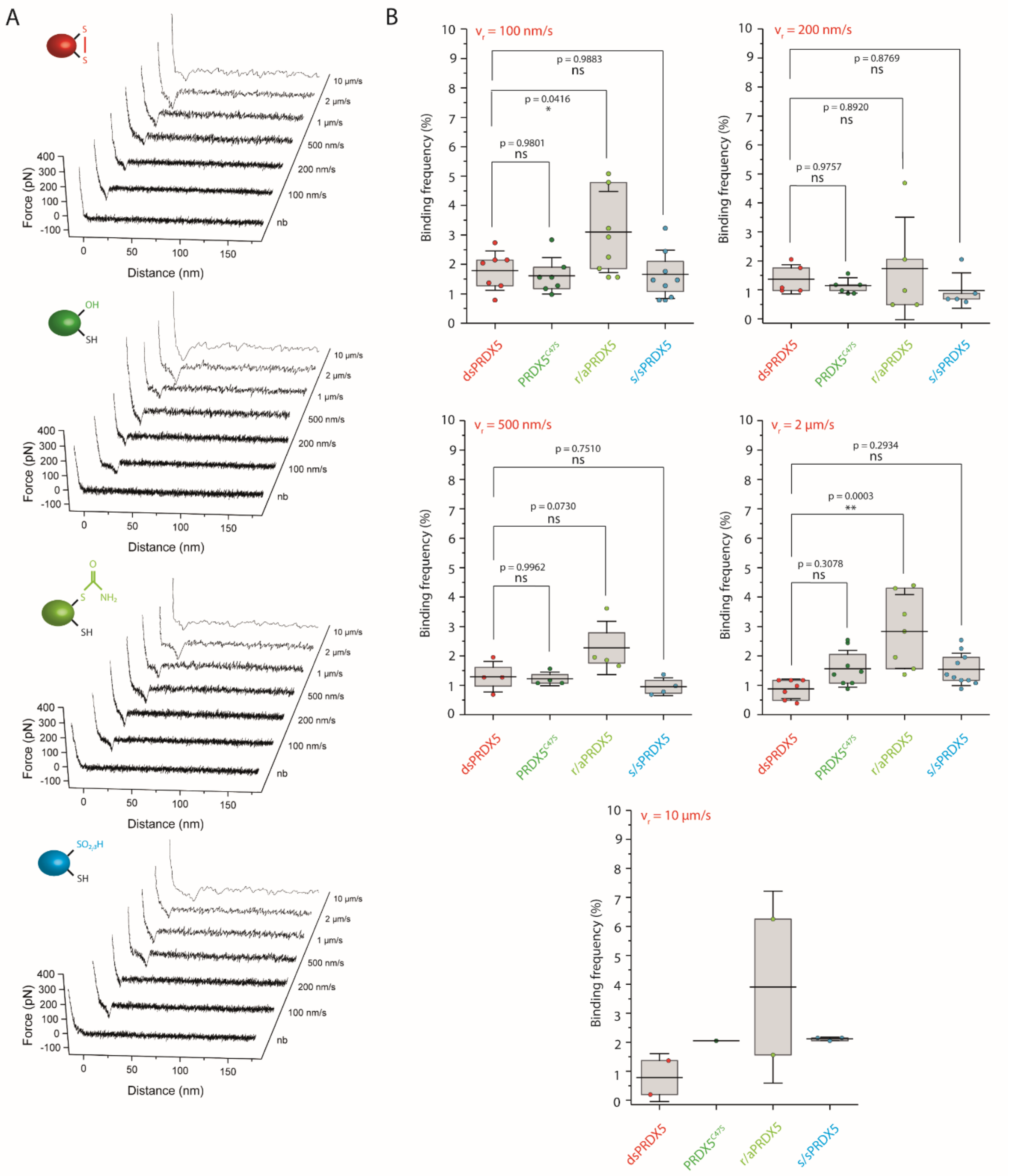

Figure S5. SMFS analysis of PRDX5 redox form binding to surface-immobilized TLR4. (A) Representative non-adhesive force-distance (FD) curve (nb, no binding) or FD curves showing a specific adhesive event, at the 6 tip retraction speeds, for all the PRDX5 redox forms. **(B)** Box plots showing the binding frequencies (BF) obtained at tip retraction speeds of 100 nm·s^{-1}, 200 nm·s^{-1}, 500 nm·s^{-1}, 2 µm·s^{-1}, and 10 µm·s^{-1}. Number of AFM maps (circles). Box plots show mean (×), median (–), 25th and 75th percentiles (boxes), and range from min to max (whiskers). ns, not significant ($p > 0.05$). *$p \leq 0.05$, **$p \leq 0.01$ (Tukey HSD test).

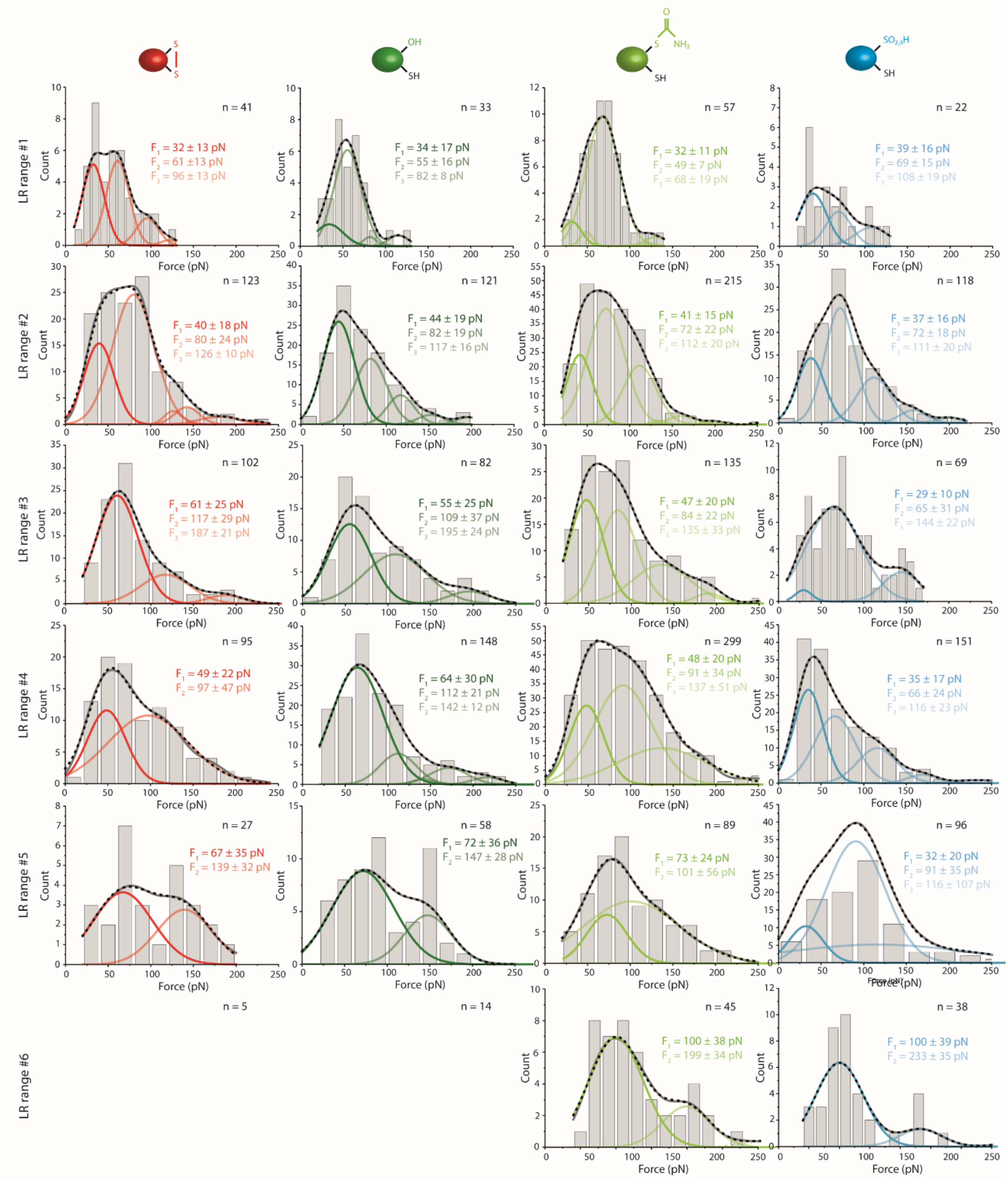

Figure S6. Kernel density function of the rupture forces obtained at the different loading rate (LR) ranges for the PRDX5 redox forms. Rupture forces were divided into discrete LR ranges (LR#1: 315-1000 pN·s^{-1}; LR#2: 1000-3150 pN·s^{-1}; LR#3: 3150-10,000 pN·s^{-1}; LR#4: 10,000-31,500 pN·s^{-1}; LR#5: 31,500-100,000 pN·s^{-1}; LR#6: 100,000-315,000 pN·s^{-1}). Kernel density function (grey line) of the rupture forces (which is superimposed on force histograms for more clarity) was then used to determine the hidden peaks based on the second derivative. The peak centers determined with the Kernel density function were then used to fit a Gaussian function (colored lines), from which the most probable rupture forces for single (1st peak) and multiple interactions (following peaks) were determined by taking peak centers (F_1, F_2, F_3 for single, double and triple interactions, respectively). The cumulative peak fit (black dashed line) overlap very well with the Kernel density function.

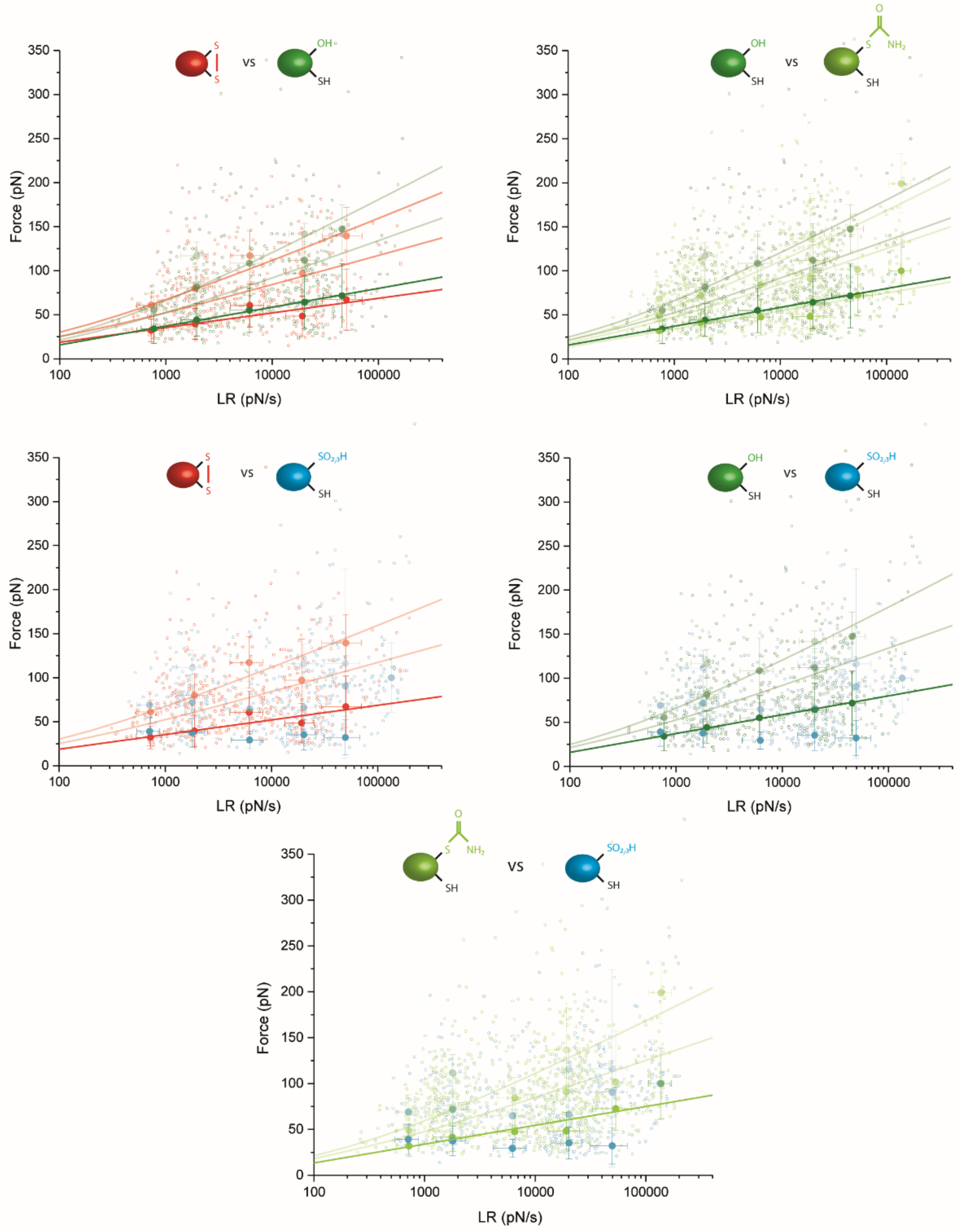

Figure S7. Superimposition of dynamic force spectroscopy (DFS) plots. PRDX^{C47S} DFS pot was superimposed with dsPRDX5, r/aPRDX5, and s/sPRDX5 DFS plots. s/sPRDX5 DFS plot was superimposed with dsPRDX5, and r/aPRDX5 DFS plots.

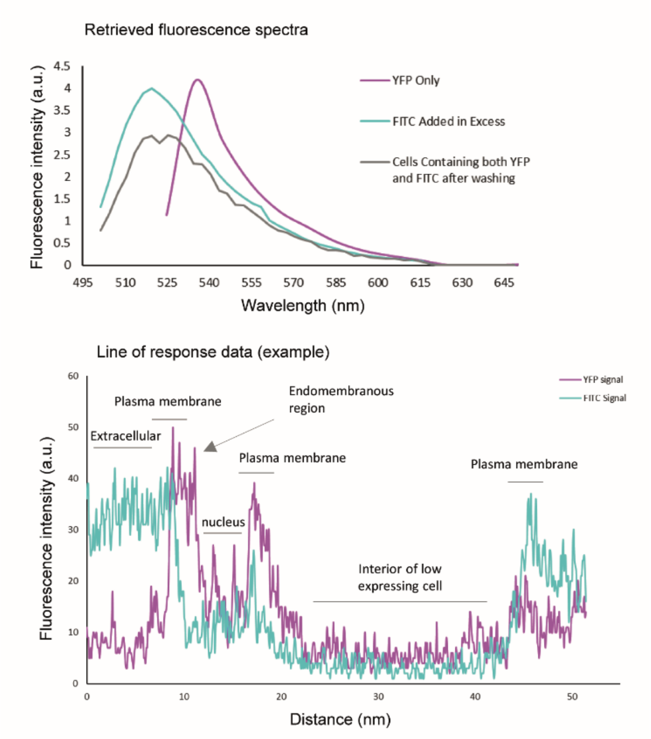

Figure S8. **Additional fluorescence data from confocal laser scanning microscopy (CLSM).** Spectral scan data retrieved from YFP (magenta) cells (upper), PRDX5-NTA-FITC (cyan) added in excess to dominate the spectra, and after YFP positive cells had been exposed and washed (gray). The shifting dual peak in the latter is indicative of more than one fluorescent species being present, although the PRDX5-NTA-FITC is the most common. Line of Response (LoR) through two adjacent cells, showing local distribution of the two fluorophores (FITC represented by cyan, YFP by magenta). FITC fluorescence intensity is highest at the edge of the plasma membrane and in the surrounding extracellular medium, whereas YFP predominates at the plasma membrane and in the endomembranous region (target site and manufacturing locale respectively). The co-occurring peaks support the co-localization analysis presented in the main manuscript.

Table S1. Masses and intensities of the peaks corresponding to monomeric species detected by ESI-Q-TOF MS on intact proteins. Peaks ≥ 20% in intensity relatively to the largest peak were considered and taken into account for the calculation of the relative intensity. The most probable Cys modification(s) corresponding to the mass shifts are given.

	Peak #	Intensity	Relative intensity (%)	Mass (Da)	Identification	Mass shift (Da)
**dsPRDX5**	1	6.11×10^{9}	27	18,294.93	Disulfide Cys-S-S-Cys	-2
	2	3.46×10^{9}	16	18,309.33	Disulfide Cys-S-S-Cys<br>Methylation	-2<br>+14
	3	8,.20 × 10^{9}	37	18,352.10	Disulfide Cys-S-S-Cys<br>Carbamidomethyl	-2<br>+57
	4	4.53×10^{9}	20	18,366.25	Disulfide Cys-S-S-Cys<br>Carbamidomethyl<br>Methylation	-2<br>+57<br>+14
**PRDX5^{C47S}**	1	6.01×10^{10}	79	18,337.98	Cys → Ser substitution<br>Carbamidomethyl	-16<br>+57
	2	1.63×10^{10}	21	18,351.85	Cys → Ser substitution<br>Carbamidomethyl<br>Methylation	-16<br>+57<br>+14
**r/aPRDX5**	1	5.76×10^{10}	62	18,467.74	Carbamidomethyl x 3	+57 x 3
	2	3.53×10^{10}	38	18,481.87	Carbamidomethyl x 3<br>Methylation	+57 x 3<br>+14
**s/sPRDX5**	1	1.43×10^{9}	13	18,294.64	Disulfide Cys-S-S-Cys	-2
	2	1.57×10^{9}	14	18,309.60	Disulfide Cys-S-S-Cys<br>Methylation	-2<br>+14
	3	5.46×10^{8}	5	18,325.28	?	+28.5
	4	9.73×10^{8}	9	18,342.09	Disulfide Cys-S-S-Cys<br>Sulfonic acid Cys-SO$_3$H<br>*or*<br>Sulfinic acid Cys-SO$_2$H<br>Methylation	-2<br>+48<br>*or*<br>+32<br>+14
	5	1.43×10^{9}	13	18,351.75	Disulfide Cys-S-S-Cys<br>Carbamidomethyl	-2<br>+57
	6	1.01×10^{9}	9	18,356.77	Disulfide Cys-S-S-Cys<br>Sulfonic acid Cys-SO$_3$H<br>Methylation	-2<br>+48<br>+14
	7	2.21×10^{9}	19	18,366.67	Disulfide Cys-S-S-Cys<br>Carbamidomethyl<br>Methylation	-2<br>+57<br>+14
	8	6.60×10^{8}	6	18,382.21	Carbamidomethyl<br>?	+57<br>+28.5
	9	4.44×10^{8}	4	18,400.67	Disulfide Cys-S-S-Cys<br>Sulfonic acid Cys-SO$_3$H<br>Carbamidomethyl<br>*or*<br>Sulfinic acid Cys-SO$_2$H<br>Carbamidomethyl<br>Methylation	-2<br>+48<br>+57<br>*or*<br>+32<br>+57<br>+14
	10	5.17×10^{8}	5	18,416.33	Sulfinic acid Cys-SO$_2$H x 2<br>Carbamidomethyl<br>*or*<br>Sulfonic acid Cys-SO$_3$H<br>Carbamidomethyl<br>Methylation	+32 x 2<br>+57<br>*or*<br>+48<br>+57<br>+14
	11	5.82×10^{8}	5	18,473.96	Cys-SO$_2$H alkylation adduct of IAM x 2	+89 x 2

Table S2. Masses and intensities of the peaks corresponding to dimeric species detected by ESI-Q-TOF MS on intact proteins. Peaks ≥ 40% in intensity relatively to the largest peak were considered. The most probable Cys modification(s) corresponding to the mass shifts are given. s/sPRDX5 is not shown given the high number of possible combinations.

	Peak #	Intensity	Mass (Da)	Identification	Mass shift (Da)
**dsPRDX5**	1	5.58×10^8	36,588.98	Disulfide Cys-S-S-Cys x 2	-2 x 2
	2	2.65×10^8	36,599.69	?	+6
	3	3.08×10^8	36,618.77	Disulfide Cys-S-S-Cys x 2<br>Methylation x 2	-2 x 2<br>+14 x 2
	4	3.24×10^8	36,700.80	Carbamidomethyl x 2	+57 x 2
	5	5.21×10^8	36,703.85	Disulfide Cys-S-S-Cys x 2<br>Carbamidomethyl x 2	-2 x 2<br>+57 x 2
	6	2.32×10^8	36,717.26	Disulfide Cys-S-S-Cys x 2<br>Carbamidomethyl x 2<br>Methylation	-2 x 2<br>+57 x 2<br>+14
	7	3.38×10^8	36,732.63	Disulfide Cys-S-S-Cys x 2<br>Carbamidomethyl x 2<br>Methylation x 2	-2 x 2<br>+57 x 2<br>+14 x 2
**PRDX5^{C47S}**	1	1.27×10^9	36,675.89	Cys → Ser substitution x 2<br>Carbamidomethyl x 2	-16 x 2<br>+57 x 2
	2	6.09×10^8	36,704.23	Cys → Ser substitution x 2<br>Carbamidomethyl x 2<br>Methylation x 2	-16 x 2<br>+57 x 2<br>+14 x 2
**r/aPRDX5**	1	5.57×10^8	36,820.63	Carbamidomethyl x 4	+57 x 4
	2	7.36×10^8	36,834.58	Carbamidomethyl x 4<br>Methylation	+57 x 4<br>+14
	3	3.56×10^8	36,848.87	Carbamidomethyl x 4<br>Methylation x 2	+57 x 4<br>+14 x 2
	4	3.00×10^8	36,879.91	Carbamidomethyl x 5	+57 x 5
	5	5.20×10^8	36,935.51	Carbamidomethyl x 6	+57 x 6
	6	3.96×10^8	36,945.73	?	+351
	7	3.13×10^8	36,946.98	?	+354
	8	6.95×10^8	36,964.20	Carbamidomethyl x 6<br>Methylation x 2	+ 57 x 6<br>+14 x 2

Supplementary Materials and Methods

LC-Q-TOF-MS/MS characterization of the PRDX5 cysteine redox state

Blocking of cysteine redox states

Redox states of PRDX5 C47 in dsPRDX5 and s/sPRDX5 were blocked with 55 mM IAM (Sigma-Aldrich) for 10 min at RT in the dark to alkylate the thiols, and 5 mM 5,5-Dimethyl-1,3-cyclo-hexanedione (dimedone, Sigma-Aldrich) for 5 min at RT to trap sulfenic acids [1,2].

Preparation for in solution samples

Chloroform-methanol precipitation. 20 µg of each sample were transferred to 0.5 mL polypropylene Protein LoBind tubes (Eppendorf) and precipitated with chloroform-methanol method [3].

Solubilization before trypsin digestion. Samples were dried under vacuum with a Savant SpeedVac Concentrator (ThermoFisher). 20 µL of 100 mM triethylammonium bicarbonate (TEAB), pH 8.5 were added to the pellets for solubilization and vortexed for 30 min at RT.

In-solution trypsin digestion. Proteolysis was performed with 1 µg of sequencing grade trypsin (Promega, USA) and allowed to continue overnight at 37°C. Each sample was dried under vacuum with a Savant SpeedVac Concentrator (Thermo Fisher).

Peptide separation using nanoUPLC

Before peptide separation, the samples were dissolved in 20 µL of 0.1 % (v/v) formic acid and 2% (v/v) acetonitrile (ACN). The resulting peptide mixture was separated by reverse phase chromatography on a NanoACQUITY UPLC MClass system (Waters) working with MassLynx V4.1 (Waters) software. 200 ng of digested proteins were injected on a C18 trap column, 100Å, 5 µm, 180 µm x 20 mm (Waters), and desalted using isocratic conditions with at a flow rate of 15 µL·min^{-1} using a 99% formic acid and 1% (v/v) ACN buffer for 3 min. Peptide mixture was subjected to reverse phase chromatography on a PepMap C18 column, 100Å, 1.8 µm, 75 µm x 150 mm (Waters) for 130 min at 35°C at a flow rate of 300 nL·min^{-1} using a two part linear gradient from 1% (v/v) ACN, 0.1 % formic acid to 35 % (v/v) ACN, 0.1 % formic acid for 90 min, and from 35% (v/v) ACN, 0.1 % formic acid to 85 % (v/v) ACN, 0.1 % formic acid for 10 min. The column was re-equilibrated at initial conditions after washing for 30 min at 85% (v/v) ACN, 0.1 % formic acid at a flow rate of 300 nL·min^{-1}. For online LC-MS analysis, the nanoUPLC was coupled to the mass spectrometer through a nano-electrospray ionization (nanoESI) source emitter.

LC-Q-TOF-MS/MS Analysis (DDA)

Data Dependent Analysis (DDA) was performed on an SYNAPT G2-Si high definition mass spectrometer (Waters) equipped with a NanoLockSpray dual electrospray ion source (Waters). Precut fused silica PicoTipR Emitters for nanoelectrospray, outer diameters: 360 µm; inner diameter: 20 µm; 10 µm tip; 2.5” length (Waters) were used for samples and Precut fused silica TicoTip Emitters for nanoelectrospray (outer diameters: 360 µm, inner diameter: 20 µm, 2.5” length) (Waters) were used for the lock mass solution. The eluent was sprayed at a spray voltage of 2.8 kV with a sampling cone voltage of 25 V and a source offset of 30 V. The source temperature was set to 80°C. The cone gas flow was 20 liters·h^{-1} with a nanoflow gas pressure of 0.4 bar, and the purge gas was turned off. The SYNAPT G2Si instrument was operated in DDA (data-dependent mode), automatically switching between MS and MS2. Full scan MS and MS2 spectra (m/z 400 - 2000) were acquired from 2 min after injection to 30 min in resolution mode (20,000 resolution FWHM at m/z 400) with a scan time of 0.1 sec. Tandem mass spectra of up to 10 precursors were generated in the trapping region of the ion mobility cell by using a collision energy ramp from 17/19 V (low mass, start/end) to up to 65/75 V (high mass,

start/end). Charged ions (+2, +3, +4) were selected to be submitted to the MSMS fragmentation over the m/z range from 50 to 2000 with a scan time of 0.25 sec. For the post-acquisition lock mass correction of the data in the MS method, the doubly charged monoisotopic ion of [Glu1]-fibrinopeptide B was used at 100 fmol·μL^{-1} using the reference sprayer of the nanoESI source with a frequency of 30 s at 0.5 $\mu L \cdot min^{-1}$ into the mass spectrometer.

ESI-QTOF data processing

Data were processed with UNIFI (Waters) using the known PRDX5 sequence. Dimedone, carbamidomethylation, sulfenic acid, sulfinic acid, and sulfonic acid as the variable cysteine modifications, oxidation as the variable methionine modification, trypsin as the digestion enzyme were selected, and one miss cleavage was allowed.

References

1. Denoncin, K.; Vertommen, D.; Arts, I.S.; Goemans, C.V.; Rahuel-Clermont, S.; Messens, J.; Collet, J.-F. A New Role for Escherichia Coli DsbC Protein in Protection against Oxidative Stress. *J. Biol. Chem.* **2014**, *289*, 12356–12364, doi:10.1074/jbc.M114.554055.
2. Arts, I.S.; Vertommen, D.; Baldin, F.; Laloux, G.; Collet, J.-F. Comprehensively Characterizing the Thioredoxin Interactome In Vivo Highlights the Central Role Played by This Ubiquitous Oxidoreductase in Redox Control. *Mol. Cell. Proteomics MCP* **2016**, *15*, 2125–2140, doi:10.1074/mcp.M115.056440.
3. Wessel, D.; Flügge, U.I. A Method for the Quantitative Recovery of Protein in Dilute Solution in the Presence of Detergents and Lipids. *Anal. Biochem.* **1984**, *138*, 141–143, doi:10.1016/0003-2697(84)90782-6.
